# PI3K/AKT/MTOR and ERK1/2-MAPK signaling pathways are involved in autophagy stimulation induced by caloric restriction or caloric restriction mimetics in cortical neurons

**DOI:** 10.18632/aging.202805

**Published:** 2021-03-14

**Authors:** Marisa Ferreira-Marques, André Carvalho, Cláudia Cavadas, Célia A. Aveleira

**Affiliations:** 1 CNC - Center for Neuroscience and Cell Biology, University of Coimbra, Coimbra, Portugal; 2 Faculty of Pharmacy, University of Coimbra, Coimbra, Portugal; 3 CIBB - Center for Innovative Biomedicine and Biotechnology, University of Coimbra, Coimbra, Portugal

**Keywords:** aging, autophagy, cortical neurons, caloric restriction mimetics

## Abstract

Caloric restriction has been shown to robustly ameliorate age-related diseases and to prolong lifespan in several model organisms, and these beneficial effects are dependent on the stimulation of autophagy. Autophagy dysfunction contributes to the accumulation of altered macromolecules, and is a key mechanism of promoting aging and age-related disorders, as neurodegenerative ones. We have previously shown that caloric restriction (CR), and CR mimetics Neuropeptide Y (NPY) and ghrelin, stimulate autophagy in rat cortical neurons, however by unknown molecular mechanisms. Overall, we show that CR, NPY, and ghrelin stimulate autophagy through PI3K/AKT/MTOR inhibition and ERK1/2-MAPK activation. The knowledge of these kinases in autophagy regulation and the contribution to the understanding of molecular mechanism facilitates the discovery of more targeted therapeutic strategies to stimulate autophagy, which is relevant in the context of age-related disorders.

## INTRODUCTION

Aging of the cerebral cortex, a thin layer that surrounds brain tissues, is correlated with structural and functional transformations that unfailingly prompt deficits in cognitive capabilities and increased susceptibility to neurodegenerative diseases [1]. The accumulation of damaged and toxic aggregate-prone proteins has been established as a common pathological hallmark shared by many of these neurodegenerative disorders [2].

Autophagy, an intracellular degradative system that removes damaged cellular constituents and protein aggregates, is critical for cellular constituents recycling and cellular homeostasis maintenance in a wide range of species [3, 4]. Autophagy impairment leads therefore to the accumulation of molecular waste and cellular dysfunction being a major pillar of the aging process age-related diseases pathogenesis [5–7]. Strategies that promote autophagy are relevant to counteract the aging process.

Caloric restriction, an intervention in which there is a severe reduction in the calories consumed without leading to malnutrition, remains the most robust non-pharmacological longevity-promoting intervention known to delay the onset of age-related diseases and to increase lifespan, at least in part, by stimulating autophagy, in different animal species, spanning from yeast to mammals [8–13]. The anti-aging role of caloric restriction is also tied to neuroendocrine system alterations, particularly the increase of hypothalamic NPY and circulating levels of ghrelin, orexigenic peptides which regulate food intake and energy expenditure [14–17].

Previously, we reported the ability of caloric restriction mimetic cell medium to promote autophagy in primary rat cortical neuron cultures, through the activation of NPY or ghrelin receptors signaling [18]. Moreover, our work further showed that NPY and ghrelin can replicate, *per se,* these effects, reinforcing their role as caloric restriction mimetics candidates and act as rejuvenating factors [18, 19]. Notwithstanding, the molecular pathways underlying caloric restriction and both NPY and ghrelin-induced autophagy in the cerebral cortex are not fully understood. Autophagy usually occurs through the canonical pathway, which depends on the inhibition of mammalian target of rapamycin complex 1 (mTORC1), but other pathways exist [20]. Focusing on other signaling pathways that regulate autophagy in mammalian cells, one mechanism by which growth factor signaling regulates MTOR is through the phosphatidylinositol 3-kinase (PI3K)/protein kinase B (AKT), PI3K/AKT pathway. In addition to PI3K/AKT signaling, autophagy may also be regulated by the action of the kinase (ERK)1/2 mitogen-activated protein kinase (MAPK), ERK1/2-MAPK signaling transduction cascades [21].

To this end, the aim of the present work was, therefore, to explore the intracellular signaling pathways underlying in the stimulation of autophagy by caloric restriction, and NPY and ghrelin, putative caloric restriction mimetics, in primary rat cortical neuron cultures. This work will provide new insights on the induction of autophagy by multiple kinases with special highlight on PI3K/AKT and ERK1/2-MAPK.

## RESULTS

### Caloric restriction, NPY, and ghrelin induce autophagy trough PI3K/AKT/MTOR pathway

**Figure 1:**
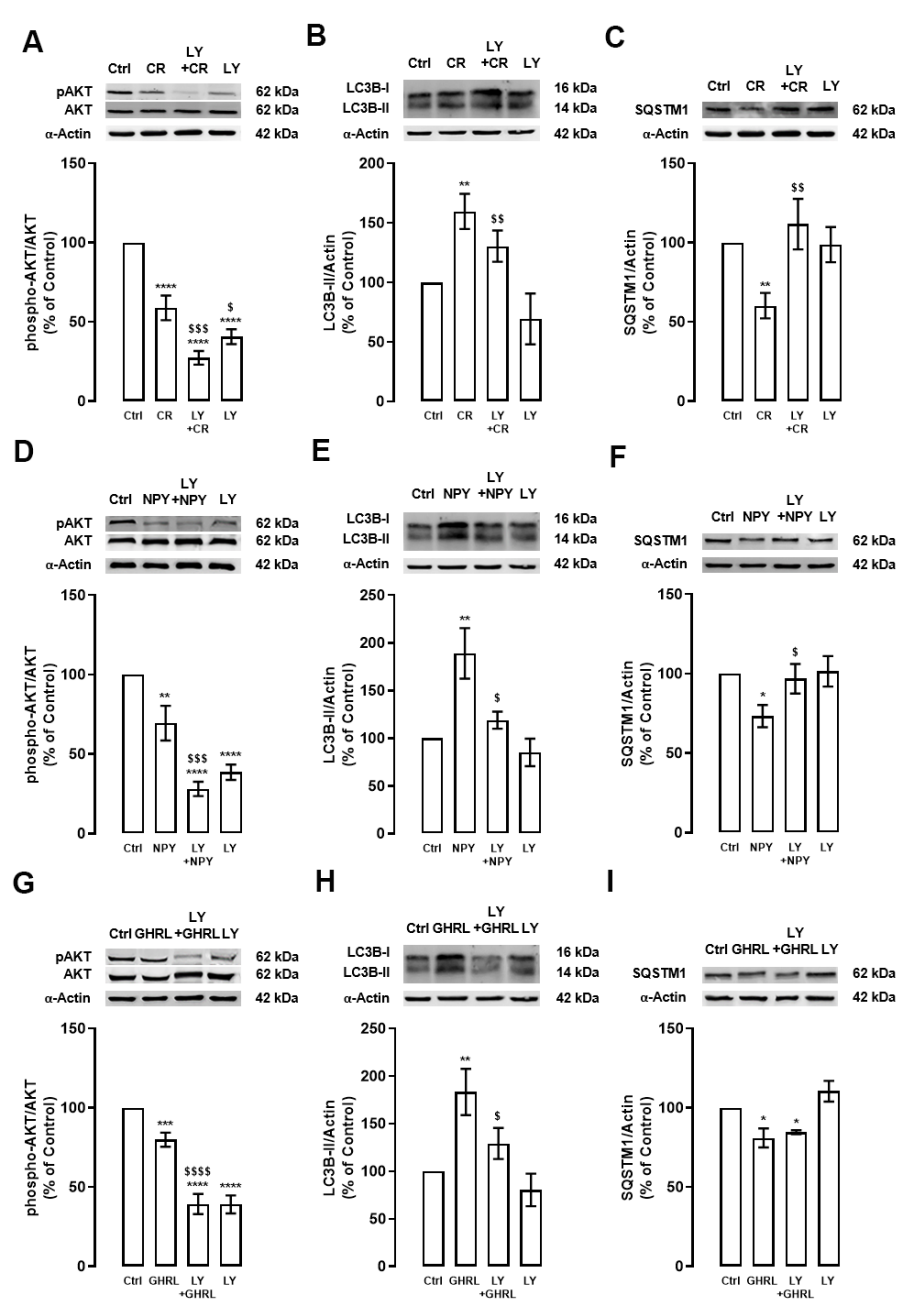
**The stimulatory effect of caloric restriction, NPY, and ghrelin on autophagy in cortical neurons is mediated by the inhibition of PI3K/AKT.** Primary rat cortical neurons were exposed to caloric restriction mimic medium (CR) - DMEM low glucose, NPY (100 nM) or ghrelin (GHRL, 10 nM) for 6 h. Untreated cells were used as control (Ctrl). (**A**–**I**) Cells were incubated with PI3K/AKT inhibitor (LY294002 (LY), 1 μM), 30 min before caloric restriction, NPY, or ghrelin treatment. Whole-cell extracts were assayed, phospho-AKT (**A**, **D**, **G**), LC3B-II (**B**, **E**, **H**), SQSTM1 (**C**, **F**, **I**) and AKT or α-Actin (loading control) immunoreactivity through Western blotting analysis, as described in Materials and Methods. Representative Western blots for each protein are presented above each respective graph. The results represent the mean±SEM of, at least, four independents experiments, and are expressed as a percentage of control. *p<0.05, **p<0.01, ***p<0.001 and ****p<0.0001, significantly different compared to control; ^$^p<0.05, ^$$^p<0.01, ^$$$^p<0.001 and ^$$$$^p<0.0001, significantly different from stimulus-treated cells, as determined by ANOVA, followed by Newman-Keuls multiple comparison test.

We evaluated the mechanisms underlying autophagy induction by caloric restriction, NPY, and ghrelin in rodent cortical neurons in culture. mTORC1 is the key molecular switch for autophagy induction. When this conserved serine/threonine kinase is inhibited, the initiation of the autophagic process is relieved and autophagy is activated [22–24]. As we have previously shown, caloric restriction stimulates autophagy in primary rat cortical neuron cultures via the canonical inhibition of MTOR activity [18]. Nonetheless, autophagy in mammalian cells is known to be regulated by several other pathways [21]. In line with this, to scrutinize the potential involvement of PI3K/AKT signaling pathway in caloric restriction-induced autophagy in cortical neurons, primary rat cortical neuron cultures were subjected to caloric restriction mimetic media (referred to as caloric restriction hereafter) for 6 h in the absence or presence of PI3K inhibitor (LY294002 (LY), 10 μM). The levels of phosphorylated AKT, a downstream effector of PI3K, and LC3B-II and SQSTM1, autophagy markers, were evaluated by Western blotting. First, as showed in Figure 1A, caloric restriction decreased AKT phosphorylation in primary rat cortical neurons and, this effect was exacerbated by the PI3K inhibitor. As expected, PI3K inhibitor alone significantly decreased AKT phosphorylation. Regarding autophagy, caloric restriction increased LC3B-II levels in primary rat cortical neurons and this effect was partially blocked by PI3K inhibitor (Figure 1B). Concomitant with LC3B-II increase, caloric restriction decreased SQSTM1 protein content and PI3K inhibitor inhibited this effect (Figure 1C). These data suggest that PI3K/AKT intracellular signaling pathway inhibition is necessary for caloric restriction-induced autophagy in primary rat cortical neuron cultures. However, other signaling pathways may be modulated under caloric restriction to regulate autophagy.

Primary rat cortical neuron cultures were treated with 100 nM NPY or 10 nM ghrelin in the absence or presence of LY (10 μM), to support that NPY and ghrelin act as caloric restriction mimetics candidates, as we previously demonstrated [18]. After 6 h, the levels of phosphorylated AKT and, LC3B-II and SQSTM1, autophagic markers, were evaluated by Western blotting. NPY and ghrelin decreased AKT phosphorylation in rat cortical neuron cultures (Figure 1D, 1G), as observed in caloric restriction condition (Figure 1A), and these effects were exacerbated by the PI3K inhibitor. Similar to caloric restriction, the increase in LC3B-II levels promoted by NPY and ghrelin (Figure 1E, 1H, respectively) was abolished by the PI3K/AKT inhibitor (Figure 1E, 1H). These results suggest that NPY and ghrelin, like caloric restriction, induce autophagy via inhibition of PI3K/AKT intracellular signaling pathway. As shown in Figure 1F, 1I, SQSTM1 levels decreased upon NPY and ghrelin treatment, respectively. However, while PI3K inhibitor blocked NPY effect, it had no effect on ghrelin-induced decrease in SQSTM1 protein levels. PI3K alone had no impact on the levels of both autophagy markers (Figure 1E, 1H, 1F, 1I).

**Figure 2:**
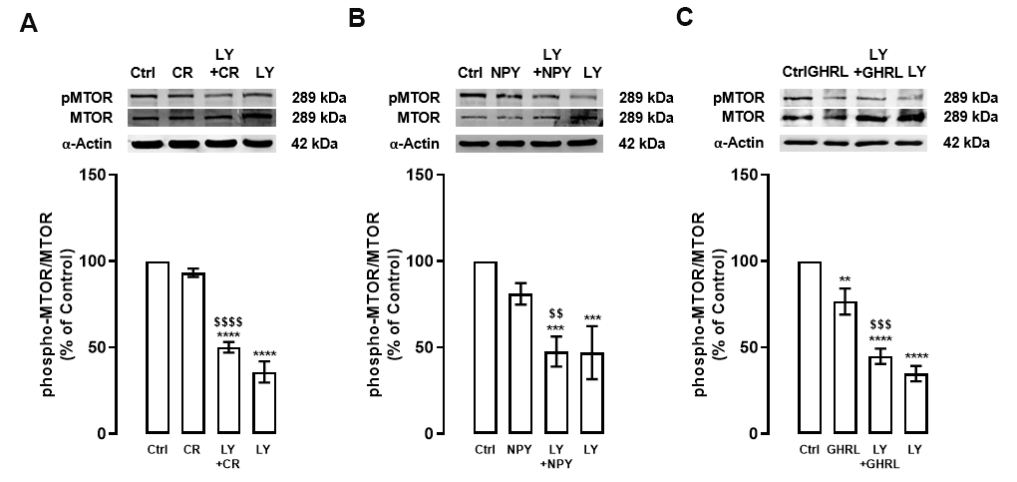
**PI3K inhibition decreases MTOR activity in primary rat cortical neurons.** Primary rat cortical neurons were exposed to caloric restriction mimic medium (CR) - DMEM low glucose, NPY (100 nM), or ghrelin (GHRL, 10 nM) for 6 h. Untreated cells were used as control (Ctrl). (**A**–**C**) Cells were incubated with PI3K/AKT inhibitor (LY294002 (LY), 1 μM), 30 min before caloric restriction, NPY, or ghrelin treatment. Whole-cell extracts were assayed, phospho-MTOR (**A**–**C**), and MTOR or α-Actin (loading control) immunoreactivity through Western blotting analysis, as described in Materials and Methods. Representative Western blots for each protein are presented above each respective graph. The results represent the mean±SEM of, at least, four independents experiments, and are expressed as a percentage of control. **p<0.01, ***p<0.001 and ****p<0.0001, significantly different compared to control; ^$$^p<0.01, ^$$$^p<0.001 and ^$$$$^p<0.0001, significantly different from stimulus-treated cells, as determined by ANOVA, followed by Newman-Keuls multiple comparison test.

Considering these observations and to better understand the involvement of PI3K/AKT pathway on the regulation of autophagy machinery by caloric restriction and NPY and/or ghrelin, we investigated the effects of PI3K inhibitor on MTOR, a downstream effector of this pathway, under caloric restriction or caloric restriction mimetics in primary rat cortical neurons. As shown in Figure 2, similarly to AKT, the levels of phosphorylated MTOR were reduced after caloric restriction (Figure 2A), or NPY and ghrelin (Figures 2B, 2C), and this effect was exacerbated in the presence of PI3K inhibitor. As expected, PI3K inhibitor alone decreased the activity of MTOR (Figure 2). These findings indicate that caloric restriction and NPY and ghrelin enhance autophagy through MTOR inhibition, although other signaling pathways may be involved.

Overall, we show that PI3K/AKT/MTOR intracellular signaling cascade inhibition, is necessary for caloric restriction- and NPY/ghrelin-induced stimulation of autophagic process in primary rat cortical neuron cultures further supporting NPY and ghrelin as caloric restriction mimetics.

### Caloric restriction, NPY, and ghrelin induce autophagy trough ERK1/2-MAPK pathway

We then investigated other possible molecular mechanisms involved in caloric restriction-, NPY-, and ghrelin-induced autophagy. Rat cortical neuron cultures were exposed to caloric restriction, NPY (100 nM), or ghrelin (10 nM) for 6 h in the absence or presence of ERK1/2-MAPK inhibitor (U0126 (U0), 10 μM). The levels of ERK phosphorylation and autophagic markers, LC3B-II, and SQSTM1, were evaluated by Western blotting.

**Figure 3:**
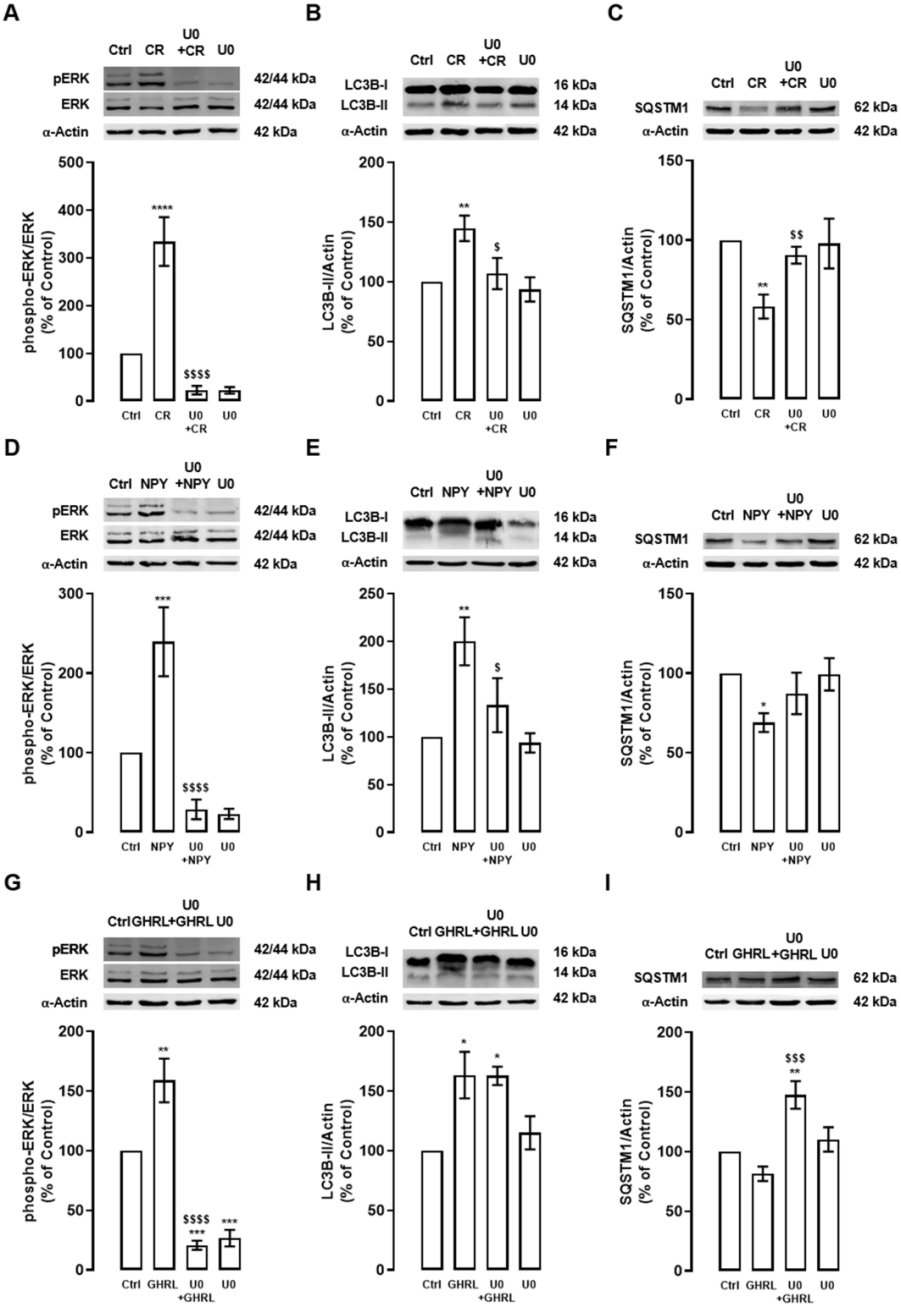
**The stimulatory effect of caloric restriction, NPY, and ghrelin on autophagy in cortical neurons is mediated by the activation of ERK1/2-MAPK.** Primary rat cortical neurons were exposed to caloric restriction mimic medium (CR) - DMEM low glucose, NPY (100 nM), or ghrelin (GHRL, 10 nM) for 6 h. Untreated cells were used as control (Ctrl). (**A**–**I**) Cells were incubated with ERK1/2-MAPK inhibitor (U0126 (U0), 1 μM), 30 min before caloric restriction, NPY or ghrelin treatment. Whole-cell extracts were assayed, phospho-ERK (**A**, **D**, **G**), LC3B-II (**B**, **E**, **H**), SQSTM1 (**C**, **F**, **I**), and ERK or α-Actin (loading control) immunoreactivity through Western blotting analysis, as described in Materials and Methods. Representative Western blots for each protein are presented above each respective graph. The results represent the mean±SEM of, at least, four independents experiments, and are expressed as a percentage of control. *p<0.05, **p<0.01, ***p<0.001 and ****p<0.0001, significantly different compared to control; ^$^p<0.05, ^$$^p<0.01, ^$$$^p<0.001 and ^$$$$^p<0.0001, significantly different from stimulus-treated cells, as determined by ANOVA, followed by Newman-Keuls multiple comparison test.

First, as showed in Figure 3A, caloric restriction increased ERK phosphorylation in primary rat cortical neuron cultures and, as expected, ERK1/2-MAPK inhibitor blocked this effect. Caloric restriction-induced autophagy, as shown by the increase in LC3B-II and decrease in SQSTM1 protein levels, was inhibited by ERK1/2-MAPK inhibitor (Figure 3B, 3C). These observations suggest that ERK1/2-MAPK intracellular signaling cascade activation is necessary for caloric restriction-induced autophagy in primary rat cortical neuron cultures.

NPY and ghrelin, as caloric restriction, also increased ERK phosphorylation in primary rat cortical neurons, and this effect was blocked by ERK1/2-MAPK inhibitor (Figure 3D, 3G). NPY, like caloric restriction, increased LC3B-II and decrease SQSTM1 levels and, this effect was partially blocked by ERK1/2-MAPK inhibition (Figure 3E, 3F). Interestingly, while upon ghrelin treatment no alterations were observed in LC3B-II levels, in the presence of ERK1/2-MAPK inhibitor, SQSTM1 levels increased (Figure 3H, 3I), suggesting that the inhibition of these pathway blocks SQSTM1 degradation.

These results might explain that ERK1/2-MAPK intracellular signaling pathway activation is implicated in the caloric restriction- and NPY-induced autophagy in rat cortical neuron cultures. The results also suggest that ghrelin-induced autophagic degradation is impaired under ERK1/2-MAPK inhibition.

### Caloric restriction, NPY, and ghrelin induce autophagy: crosstalk between PI3K/AKT and ERK1/2-MAPK

To further dissect the mechanisms involving both kinases, PI3K/AKT and ERK1/2-MAPK intracellular signaling cascades, in the regulation of autophagy, we further explore the interplay between these two signaling pathways in primary rat cortical neurons.

**Figure 4:**
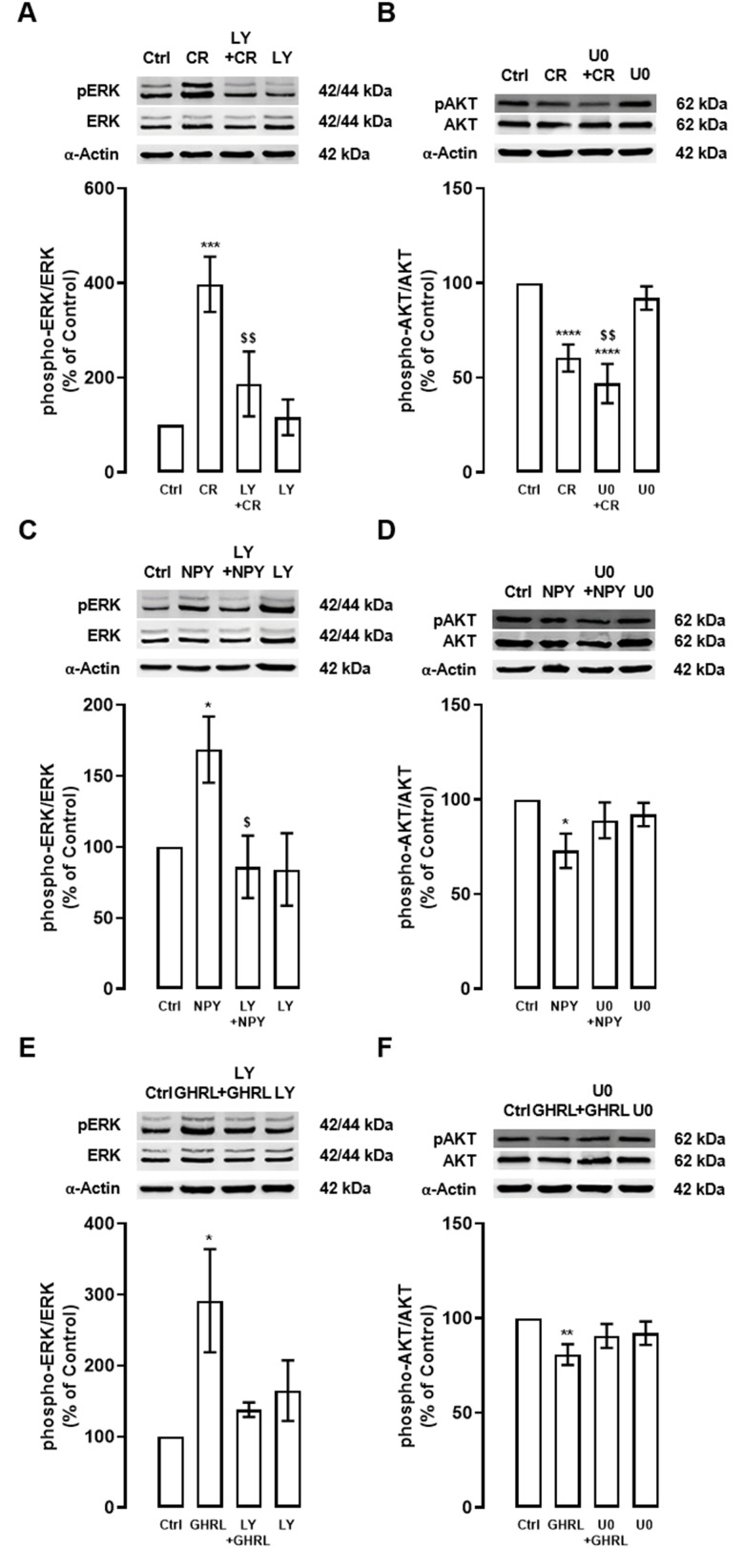
**Interplay between PI3K/AKT and ERK1/2-MAPK signaling pathways under caloric restriction, NPY, and ghrelin-induced autophagy in cortical neurons.** Primary rat cortical neurons were exposed to caloric restriction mimic medium (CR) - DMEM low glucose, NPY (100 nM), or ghrelin (GHRL, 10 nM) for 6 h. Untreated cells were used as control (Ctrl). (**A**–**F**) Cells were incubated with PI3K/AKT inhibitor (LY294002 (LY), 1 μM) or ERK1/2-MAPK inhibitor (U0126 (U0), 1 μM), 30 min before caloric restriction, NPY, or ghrelin treatment. Whole-cell extracts were assayed, phospho-ERK (**A**, **C**, **E**) and phospho-AKT (**B**, **D**, **F**) and ERK or AKT (loading control) immunoreactivity through Western blotting analysis, as described in Materials and Methods. Representative Western blots for each protein are presented above each respective graph. The results represent the mean±SEM of, at least, four independents experiments, and are expressed as a percentage of control. *p<0.05, **p<0.01, ***p<0.001 and ****p<0.0001, significantly different compared to control; ^$^p<0.05 and ^$$^p<0.01, significantly different from stimulus-treated cells as determined by ANOVA, followed by Newman-Keuls multiple comparison test.

Caloric restriction increased ERK phosphorylation and PI3K/AKT inhibitor (LY) inhibited the ERK1/2-MAPK signaling pathway (Figure 4A). Interestingly, we observed that caloric restriction decreased AKT phosphorylation and ERK1/2-MAPK inhibitor (U0) had no effect on AKT phosphorylation (Figure 4B). These observations pinpoint that AKT is upstream of ERK in the caloric-restriction-induced stimulation of autophagy.

Similarly to caloric restriction, as shown in Figure 4C, 4E, both NPY and ghrelin increased ERK phosphorylation and this effect was inhibited by PI3K/AKT inhibitor (LY). We also observed that NPY decreased AKT phosphorylation and ERK1/2-MAPK (U0) inhibitor partially reversed this effect (Figure 4D). Interestingly, we also observed that ghrelin decreased AKT phosphorylation and ERK1/2-MAPK inhibitor seems to revert this effect (Figure 4F). Overall, these findings suggest that these two pathways are at the same level, but are not independent of each other, in the mechanism of autophagy induction by NPY and ghrelin.

## DISCUSSION

Here, we provide new evidence that caloric restriction, and NPY and ghrelin, caloric restriction mimetics, stimulate autophagy through PI3K/AKT/MTOR inhibition and ERK1/2-MAPK activation in cortical neurons.

The capability of healthy cells to efficiently dealt with dysfunctional cellular and molecular components rely on autophagy induction as an adaptive response to stress, allowing it to maintain cell survival under nutrient starvation or clear protein aggregates, malfunction organelles or, intracellular pathogens [25–27]. Autophagy decline has been associated with aging and age-related diseases, therefore restoring autophagic capacity of cells has been considered an attractive therapeutic target to delay aging and promote health [28–30]. The main mechanisms that control autophagy induction are already known. The canonical autophagy pathway consists in the inactivation of the mTORC1, a nutrient-sensor signaling pathway known to regulate longevity [31]. It is a well-known molecular switch for autophagy induction, but there are other signal transduction cascades that regulate autophagy downstream of MTOR, at known or yet unknown points. Overall, there is still much to uncover regarding the factors and signaling pathways regulating the induction of autophagic process in mammalian cells, as well as some disparity, may be related to cell-type specificity, which still need to be addressed.

As we previously reported, caloric restriction, NPY, and ghrelin induce autophagy and also decrease MTOR phosphorylation, suggesting that caloric restriction and caloric restriction mimetics induce autophagy in primary rat cortical neuron cultures through mTORC1 inhibition [18]. This was an expected outcome, given the fact that MTOR signaling responds to different intra- and extracellular conditions [20]. However, as the primary rat cortical neuron cultures used were under nutrient-rich conditions, some MTOR kinase activity is still observed, suggesting that other signaling pathways may regulate autophagy independent of canonical MTOR inhibition as investigated in the present paper.

Bearing in mind that canonical autophagy pathway, MTOR, acts as a crucial regulator of the balance between the autophagic process and growth, it is understandable that other cell signaling pathways are interconnected with MTOR and are responsible for the orchestration of the metabolic regulation of the cells according to the extracellular conditions [23]. Phosphatidylinositol-3-kinase, PI3K/AKT, is the canonical cascade that negatively controls autophagy, through MTOR activation. PI3K/AKT inhibitors, such as 3-methylladenine, wortmannin, and LY294002, have, therefore, been used as autophagy inhibitors. Once used, these compounds block class I as well as class III PI3Ks. While class III PI3Ks are required for the initiation and progression of autophagy, class I PI3Ks indirectly activate MTOR complex and suppress the autophagic turnover pathway. In sum, the net effect of these pharmacological inhibitors is commonly to inhibit autophagy due to the class III enzymes, which are needed to switch-on autophagy, acting downstream of the negative regulatory class I [32]. In contrast, a kinase (ERK)1/2 mitogen-activated protein kinase (MAPK), ERK1/2-MAPK, has also been linked to autophagy regulation [33, 34]. ERK signaling pathway and initiation of autophagosome formation at the plasma membrane, suggesting a potential functional association between components of the ERK signaling transduction cascade and autophagosome [35, 36]. Nevertheless, there is still a knowledge gap on the autophagy machinery regulation by these kinases.

We investigated the role of kinase signaling cascades, PI3K/AKT and ERK1/2-MAPK, on autophagy induction upon caloric restriction, NPY, and ghrelin exposure in rat cortical neuron cultures. By using selective inhibitors of the identified pathways, we show that both pathways are involved, at least in some steps of the autophagic process, for the three conditions. Our study is significant in demonstrating the functional interplay between the kinases under this study, PI3K/AKT and ERK1/2-MAPK, in promoting autophagy *in vitro*. Our results show that autophagy induced by caloric restriction is dependent on the inhibition of AKT and activation of ERK signaling pathways. Moreover, our results also suggest that AKT is upstream of ERK pathway in this regulatory process. On the other hand, we report that AKT and ERK pathways act independently from each other in the NPY and ghrelin stimulation of autophagy. The knowledge of kinases involved in autophagy regulation, and the deciphering of the precise mechanisms at the molecular within this machinery, might facilitate the development of more targeted pharmaceutical approaches and provide new putative therapeutic tools to stimulate autophagy, which is relevant in the context of age-related deteriorations and extend longevity.

Considering autophagy as a major mechanism underlying caloric restriction’s beneficial effects and regarding that its application for long periods in humans is very difficult to maintain, the discovery of caloric restriction mimetic strategies that induce the beneficial effect of caloric restriction without its discomfort are very relevant. Our studies reinforce, that NPY and ghrelin are putative caloric restriction mimetic candidates. Besides aging is correlated with reduced levels of NPY and ghrelin and decreased autophagic function, it is tempting to speculate a potential link between the beneficial effect of NPY and ghrelin on autophagy and the delay of aging. Unravelling the key mechanism(s) underlying the autophagy induction in young and healthy cells is crucial to develop caloric restriction mimetics and more targeted therapeutic approaches that exploit hormone- and nutrient-sensitive signaling circuitries and to turn back the time.

## MATERIALS AND METHODS

### Animals

For all experiments in this work, female Wistar rats were acquired from Charles River Laboratories (L'Arbresle, France). Experiments were performed in accordance with the guidelines of the European Union Directive for Animal Research, Directive 86/609/EEC for the use and care of animals in the laboratory, translated to the Portuguese law in 2013 (Decreto-lei 113/2013). All researchers involved in this study received competent training [(Federation of Laboratory Animal Science Associations (FELASA)-certified course] and certification to perform the experiments from the Portuguese authorities (Direcção Geral de Alimentação e Veterinária). The present work and described animal experimentation was approved by the Portuguese Science Foundation. CNC – Center for Neuroscience and Cell Biology – University of Coimbra animal experimentation board approved the utilization of animals for this project (reference PTDC/SAU-FCF/099082/2008). The number of animals used was minimized to reduce animal suffering.

### Primary rat cortical neuron cultures

Primary rat cortical neuron cultures were obtained as previously published by our group [18]. In short, cortical neurons were obtained from brain rats at embryonic days 18-19 (E18-19) and cultured in high glucose (4.5 g.L^-1^) Neurobasal medium with 500 μM L-Glutamine, 2 % B27 supplement, 100 U.mL^-1^ penicillin and, 100 μg.mL^-1^ streptomycin (all from Gibco), with no growth factors, on poly-D-Lysine coated cell culture plates. Cortical neurons were maintained at 37° C in a humidified incubator with 5 % CO_2_/air for 8 days and the medium was replaced every fourth day by aspirating half of the medium from each well and replacing it with fresh medium.

### Experimental conditions

To mimic a caloric restriction condition, primary rat cortical neuron cultures were exposed to a Dulbecco’s Modified Eagle Medium (DMEM; Sigma- Aldrich) low glucose medium (1 g.L^-1^ glucose, 100 U.mL^-1^ penicillin and, 100 μg.mL^-1^ streptomycin, without B27 supplementation) or, NPY (100 nM; Phoenix Europe GmbH) or acylated ghrelin (10 nM; Bachem) as previous described [18]. Protein kinase inhibitors were used as follow: PI3K/AKT inhibitor (LY294002; 10 μM) and ERK1/2-MAPK inhibitor (U0126; 10 μM), both from Merck Millipore. Inhibitors were added to cell culture medium 30 min before the addition of caloric restriction mimetics NPY or ghrelin for 6 h.

### Western blotting

Western blotting of rat cortical neurons whole-cell lysates was performed as previously described by our group [18]. Briefly, cell lysates were separated by electrophoresis in SDS-PAGE and, transferred electrophoretically to PVDF membranes (Millipore). After blotting, the membranes were blocked in 5 % non-fat milk in Tris-buffered saline containing 0.1 % Tween 20 (TBS-T) for one hour at room temperature and then incubated overnight with the primary antibodies at 4° C. The primary antibodies used were: rabbit polyclonal anti-LC3B, anti-SQSTM1, anti-phospho-AKT (Ser^473^), anti-AKT, anti-phospho-p44/42 MAPK (MEK/ERK1/2) (Thr^202^/Tyr^204^), anti-p44/42 MAPK (MEK/ERK1/2), anti-phospho-MTOR (Ser^2448^) and anti-MTOR, all at a dilution 1:1000, from Cell Signaling Technologies. Thereafter, the membranes were incubated with alkaline phosphatase-linked secondary antibodies, specific to rabbit IgG or mouse IgG in a 1:10000 dilution (GE Healthcare). Protein immunoreactivity was detected by chemifluorescence using the ECF substrate (GE Healthcare) in a VersaDoc Imaging System (Bio-Rad) and protein bands optical density was quantified with the Quantity One Software (Bio-Rad). Membranes were reprobed for α-Actin (Sigma; 1:10000 dilution) for equal protein loading control.

### Statistical analysis

Results are expressed as mean ± SEM. Newman-Keuls multiple comparison test following Ordinary one-way ANOVA were used to determine significant differences between control and stimulus-treated cells, as indicated in figure legends. Values of p<0.05 were considered statistically significant. GraphPad Prism for Windows (GraphPad Software, Version 8.42) was used for all statistical analyses.
